# An integrated isotopic labeling and freeze sampling apparatus (ILSA) to support sampling leaf metabolomics at a centi-second scale

**DOI:** 10.1186/s13007-022-00926-7

**Published:** 2022-07-30

**Authors:** Qiming Tang, Qingfeng Song, Xiaoxiang Ni, Zai Shi, Genyun Chen, Xinguang Zhu

**Affiliations:** 1 National Key Laboratory for Plant Molecular Genetics, CAS Center for Excellence in Molecular Plant Sciences, Shanghai Institute of Plant Physiology and Ecology, Chinese Academy of Sciences, Shanghai, 200032 China; 2 University of Chinese Academy of Sciences, Beijing, 100049 China

**Keywords:** Isotopic labeling, Freeze sampling, Chamber, Metabolism, HPLC-MS/MS, Photosynthesis

## Abstract

**Background:**

Photosynthesis close interacts with respiration and nitrogen assimilation, which determine the photosynthetic efficiency of a leaf. Accurately quantifying the metabolic fluxes in photosynthesis, respiration and nitrogen assimilation benefit the design of photosynthetic efficiency improvement. To accurately estimate metabolic fluxes, time-series data including leaf metabolism and isotopic abundance changes should be collected under precisely controlled environments. But for isotopic labelled leaves under defined environments the, time cost of manually sampling usually longer than the turnover time of several intermediates in photosynthetic metabolism. In this case, the metabolic or physiological status of leaf sample would change during the sampling, and the accuracy of metabolomics data could be compromised.

**Results:**

Here we developed an **i**ntegrated isotopic **l**abeling and freeze **s**ampling **a**pparatus (ILSA), which could finish freeze sampling automatically in 0.05 s. ILSA can not only be used for sampling of photosynthetic metabolism measurement, but also suit for leaf isotopic labeling experiments under controlled environments ([CO_2_] and light). Combined with HPLC–MS/MS as the metabolic measurement method, we demonstrated: (1) how pool-size of photosynthetic metabolites change in dark-accumulated rice leaf, and (2) variation in photosynthetic metabolic flux between rice and *Arabidopsis thaliana*.

**Conclusions:**

The development of ILSA supports the photosynthetic research on metabolism and metabolic flux analysis and provides a new tool for the study of leaf physiology.

**Supplementary Information:**

The online version contains supplementary material available at 10.1186/s13007-022-00926-7.

## Introduction

Photosynthetic metabolism interacts closely with respiration and nitrogen assimilation, which determines photosynthetic efficiency of plants [1]. There are large natural variations in photosynthetic metabolism, but the interactions between photosynthesis, respiration and nitrogen assimilation are still unclear [2–4]. There are several reports on the engineering of photorespiratory bypasses to enhance photosynthesis, which suggest the potential for manipulating the photosynthesis and associated metabolism for higher yield potential [5–7]. Physiological studies also suggest that manipulating photorespiratory fluxes may potentially increase photosynthetic rates under photorespiratory conditions [8]. Therefore, studying the natural variations of metabolic fluxes of leaves in different plants, in different cultivars of the same species, or under different environments may provide new options to improve photosynthesis for greater yield [9].

To characterize photosynthetic metabolism, information from gas exchange [10], metabolomics [11–13] and metabolic flux [3, 14–16] are all required. Among these measurements, the development of metabolic sampling methods is challenging. One of the major challenges in sampling leaf material to study leaf photosynthetic metabolism is that the response time of different photosynthetic processes to environmental variations varies from 10^–5^ to 10^4^ min [17, 18]. For example, when ambient light level changes, the chlorophyll fluorescence changes within 0.1 s [19] and electron transport rate starts to change at about 0.1 s [17], which causes changes in the concentration of ATP and NADPH and hence changes in the downstream photosynthetic metabolism. The concentrations of metabolites in Calvin-Benson cycle can be affected within 2 s after the light level is changed [11, 20]. One minute after light change, the state transition of photosystem and the activities of the Calvin-Benson cycle enzymes will also change [17]. The concentrations of metabolites involved in the photosynthetic metabolism respond to changes in light and CO_2_ levels at a time scale of from 0.1 s to several minutes [21–23], which is much shorter than the response of gene expression (30 min and longer) or protein translation (10 h and longer) [17].

Although great efforts have been made to improve protocols used for metabolite extraction and quantification [11, 13, 24, 25], the effort has been made to improve methods for metabolite sampling is insufficient. This is particularly bothersome for research in photosynthetic metabolism since many metabolites involved in the Calvin Benson cycle showed a turnover time less than 1 s [11]. Furthermore, for some reported experiments in which leaf sampling was done through self-made chamber [3, 26, 27], generally only one leaf can be sampled at one time. Given the large sample size required for flux analysis, the throughput of such sampling method becomes a limitation for metabolic flux analysis, given the large sample size required for flux analysis [28].

Isotopically nonstationary metabolic flux analysis (INST-MFA), as a well-developed metabolic flux analysis method, has been applied on higher plants in recent years [3, 15, 16, 27]. The application of INST-MFA requires the labeling leaves should keep at a metabolic steady state; this method also requires a rapid sampling time. An environmentally controllable leaf chamber can be used to meet these requirements. To our knowledge, chambers with controlled CO_2_ and light with an independent operator have been developed but the sampling progress needs to be manually completed [3, 15, 16, 27]. Here, we designed and implemented an **I**ntegrated isotopic **L**abeling and freeze **S**ampling **A**pparatus (ILSA), which can automatically complete isotopic labelling and the freeze sampling could be completed in 0.05 s. ILSA can connect multiple sampling units to achieve high-throughput labelling and sampling with reduced costs and short time.

## Results and discussions

### Characteristic time of leaf photosynthesis dynamics under changing light

**Fig. 1:**
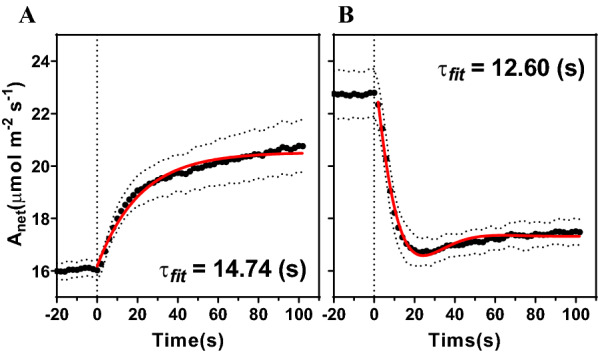
Characteristic time of leaf photosynthesis. **A** Net photosynthesis rate changed when light change from 500 μmol m^−2^ s^−1^ (− 20–0 s) to 1000 μmol m^−2^ s^−1^ (0–120 s). **B** Net photosynthesis rate changed when light change from 1000 μmol m^−2^ s^−1^ (− 20–0 s) to 500 μmol m^−2^ s^−1^ (0–120 s) Dash line indicated the standard deviation, n = 3. Solid red line is the fitting results. The fitting method of characteristic time is described in Methods

As leaf photosynthesis is assumed as a linear time-invariant systems (LTI system), the method to obtain the characteristic time of leaf photosynthesis is based on measurement of leaf photosynthesis under dynamic light. Specifically, we measured the changes of leaf photosynthesis for the rice cultivar IR64 during the switch of light from a PPFD of 500 μmol m^−2^ s^−1^ to a PPFD of 1000 μmol m^−2^ s^−1^ (low light to high light, LH) and from high light to low light (HL). Results show that the dynamics are different between two experiments. When the PPFD was increased from low to high (LH), the rate of net photosynthesis increased monotonically (Fig. 1A). When the PPFD decreased from high light to low light (HL), the rate of net photosynthesis decreased initially, then gradually increased (Fig. 1B). These results are consistent with the step response characteristics of first-order and second-order LTI systems respectively. So, the step response of a first-order LTI system is used for LH fitting:

1 $${x}_{c}\left(t\right)=1-{\mathrm{e}}^{-\frac{1}{T}t} \left(\mathrm{t}\ge 0\right)$$

where *T* is named as the characteristic time of first-order LTI systems.

2 $$\tau =T$$

The step response of a second-order system is used for HL fitting:

3 $${x}_{c}\left(t\right)=1-\frac{1}{\sqrt{1-{\xi }^{2}}}{\mathrm{e}}^{-\xi {\omega }_{n}t}\mathrm{sin}\left({\omega }_{d}t+\theta \right) (0<\upxi <1)$$

where $\xi ,\,{\omega }_{n},\,{\omega }_{d}\,\mathrm{and } \, \theta$ are parameters of the system. When $0<\upxi <1$ , the system is *underdamped*. It means that the system oscillates and converges to a new steady state after a step signal. The characteristic time of underdamped second-order LTI systems is $\frac{1}{\xi {\omega }_{n}}$.

4 $$\tau =\frac{1}{\xi {\omega }_{n}}$$

As a result, the characteristic time (${{\varvec{\tau}}}_{{\varvec{p}}}$) of LH is 14.74 s (R^2^: 0.50) (Fig. 1A), and the ${{\varvec{\tau}}}_{{\varvec{p}}}$ is 12.60 s for HL (R^2^: 0.65) (Fig. 1B). To design the sampling system, we used 10 s as the ${{\varvec{\tau}}}_{{\varvec{p}}}$ during the calculation of the maximal theoretical upper limit of the sampling time (Table 1), according to the method developed based on the Nyquist-Shannon sampling theorem (see Methods).

**Table 1:** Summary on the time criteria of photosynthetic metabolite sampling

Symbol	Description	Value
${{\varvec{\tau}}}_{{\varvec{p}}}$	Characteristic time of leaf photosynthesis	10 s
${{\varvec{\tau}}}_{{\varvec{i}}}$	Time interval of sampling (theoretical upper limit)	5 s
${{\varvec{\tau}}}_{{\varvec{s}}}$	Time of sampling (theoretical upper limit)	0.25 s
${{\varvec{\tau}}}_{{\varvec{s}}}(\mathbf{I}\mathbf{L}\mathbf{S}\mathbf{A})$	Time of sampling using ILSA	52.64 (± 8.03) ms

### Theoretical upper limit of the sampling time (${{\varvec{\tau}}}_{{\varvec{s}}}$) and the ${{\varvec{\tau}}}_{{\varvec{s}}}$ achieved with ILSA

**Fig. 2:**
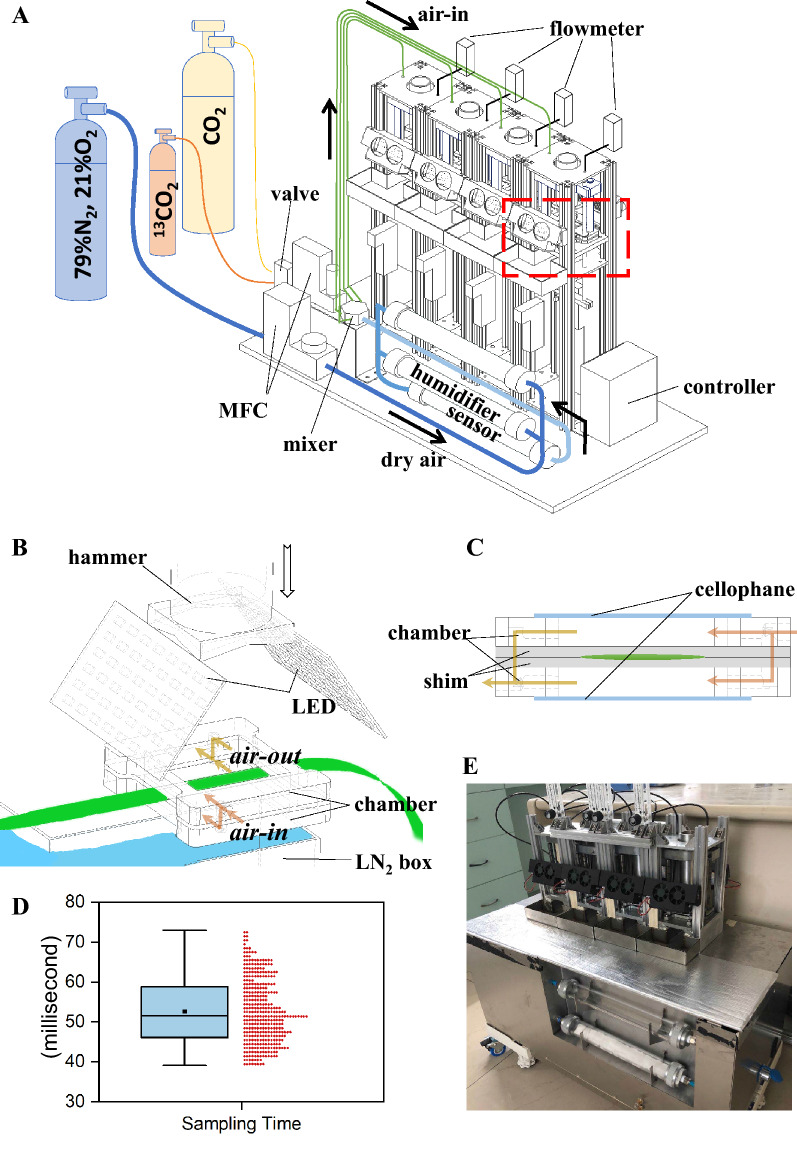
Design and implementation of ILSA. **A** Design sketch of ILSA, the part in red dotted box is zoom in and shown in **B**. **B** Sketch of freezing and sampling mechanism of ILSA. **C** Side view of semi-open chamber in ILSA. **D** Time cost of ILSA sampling. Each red dot represent one sampling test. **E** Photo of ILSA prototype

To track the change of metabolic reactions in a leaf under dynamic light environment, the time interval of sampling $({{\varvec{\tau}}}_{{\varvec{i}}}$) theoretically should not exceed 5 s, which was estimated based on Eq. 5 and the measured ${{\varvec{\tau}}}_{{\varvec{p}}}$ (Fig. 1) and the time of sampling (${{\varvec{\tau}}}_{{\varvec{s}}}$) needs to be controlled within 0.25 s (Table 1). To measure the sampling time of ILSA, we used two photoelectric switches and Raspberry Pi (Model 3B, www.raspberrypi.org) to measure the time cost for ILSA leaf sampling process. Two photoelectric switches are placed on the upper and lower sides of the cylinder respectively. The sampling time is represented by the time interval when two photoelectric switches are triggered successively. As a result, the time used during the sampling process with ILSA is 52.64 ms (S.D. 8.03 ms, n = 360, Fig. 2D). It is only 21% of the theoretical upper limit ${{\varvec{\tau}}}_{{\varvec{p}}}$. The ${{\varvec{\tau}}}_{{\varvec{p}}}$, ${{\varvec{\tau}}}_{{\varvec{i}}}$, ${{\varvec{\tau}}}_{{\varvec{s}}}$ and the ${{\varvec{\tau}}}_{{\varvec{s}}}$ achieved by ILSA are summarized in (Table 1).

### Dynamic changes of metabolite concentrations under dynamic light treatment

First, as a verification of ILSA method and metabolomic profiling protocol, rice leaves were freeze sampled and metabolites are extracted and measured following [2]. The concentration of all common measured metabolites, except glycerate, were comparable to previously published date (Additional file 1: Fig. S1), which suggests that the ILSA method and metabolomic profiling protocol can be used for metabolomics sampling.

**Fig. 3:**
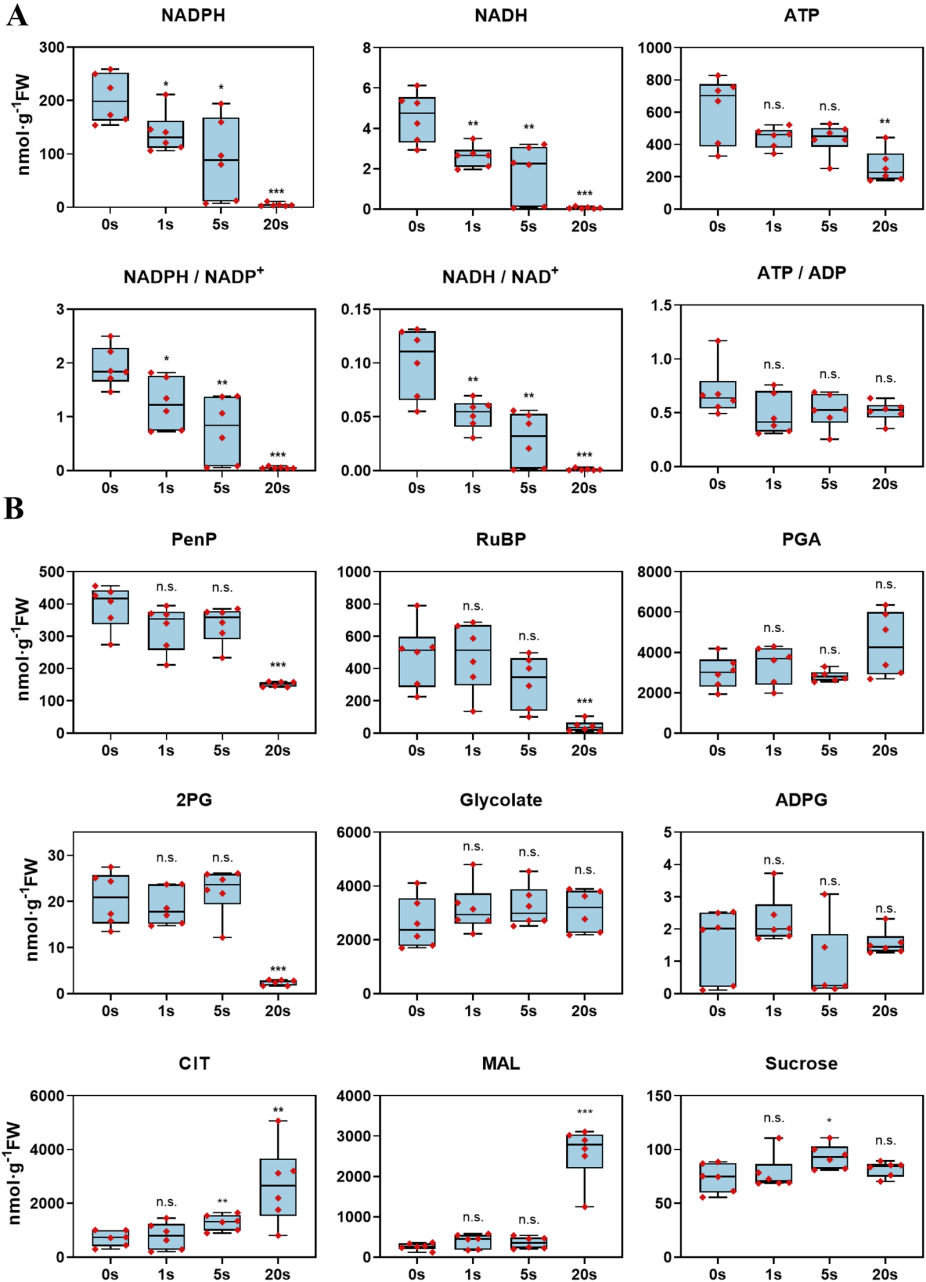
Dynamic changes of metabolite concentrations in rice under different short-dark treatments. **A** redox- and energy-related metabolites. **B** Metabolites shown a clear change pattern, for all measured metabolites, see Additional file 2: Data S1. Each black diamond in the histogram represents a sample. The significant levels of difference between 0 and 1 s, 5 s and 20 s are indicated by asterisks *p < 0.05; **p < 0.01; ***p < 0.001; n.s.: not significant

Second, we measured the dynamic changes of metabolites under a short dark treatment, all leaves were placed in ILSA and adapted under light (PAR = 1200 μmol m^−2^ s^−1^_,_ CO_2_ = 440 ppm) for 30 mins. Then, leaves were sampled after dark treatments (PAR < 5 μmol m^−2^ s^−1^) for 0, 1 s, 5 s and 20 s. The concentration of NADPH ([NADPH]) gradually decreased with the duration of the darkness. The [NADPH] was already 68.1% (*p* = 0.024, Student t-test), 44.9% (*p* = 0.11) and 2.3% (*p* < 0.0001) of the original value after 1 s, 5 s and 20 s under dark, respectively (Fig. 3A). The concentration of NADH, NADPH:NADP^+^ ratio and NADH:NAD^+^ ratio similarly decreased with the progression of dark treatment (Fig. 3A). The concentration of ATP and ATP:ADP ratio did not change significantly (Fig. 3A). The concentrations of most intermediates in the Calvin-Benson cycle decreased after leaves were kept under dark for 20 s (Fig. 3B, Additional file 3: Data S2). The concentrations of most photorespiratory intermediates were stable, except 2-PG (Fig. 3B, Additional file 3: Data S2). The concentration of citrate increased (*p* = 0.006) after 5 s under dark, and while the concentration of malate (MAL) accumulated after 20 s under dark (Fig. 3B). Concentrations of sucrose and ADPG were stable under dark. To summarize, our data show that even within one second after leaves are kept under dark, substantial changes in concentrations of many metabolites were observed, suggesting that to faithfully reflect the photosynthetic metabolic status of leaves, it is necessary to complete the sampling within 1 s, which is consistent with our earlier theoretical analysis (Table 1).

### Measuring metabolic fluxes of a photosynthetic rice leaf

**Fig. 4:**
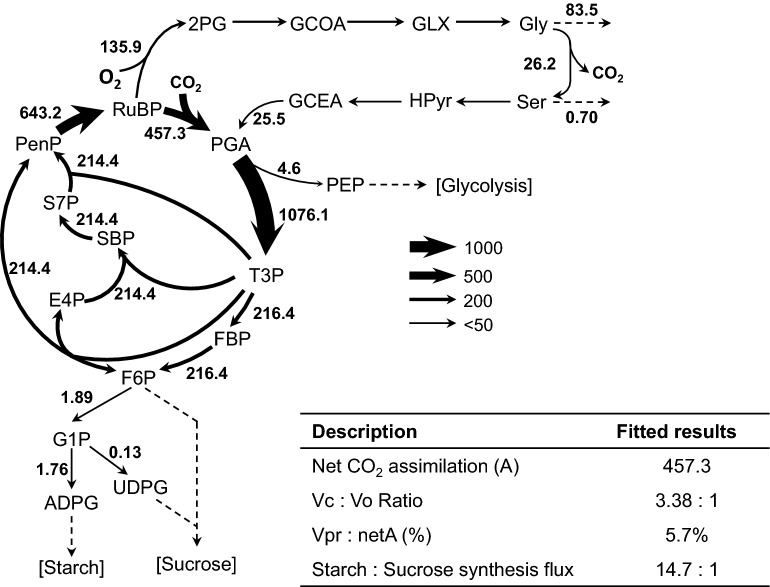
Carbon assimilatory fluxes of a photosynthetic rice leaf. **A** Rice (IR64) net flux maps sampled using ILSA. Light: 1200 μmol m^−2^ s^−1^, CO_2_:500 ppm. Absolute fluxes (nmol metabolite·gFW^−1^ s^−1^) shown. See Additional file 6: Data S5 for all flux results. B) Summary of fitted photosynthetic parameters; net CO_2_ assimilation is in terms of absolute fluxes (nmol gFW^−1^ s^−1^). Vc, carboxylation flux; Vo, oxygenation flux; netA, net CO_2_ assimilation. Vpr, photorespiratory CO_2_ release

We further analyzed the metabolic flux of the Calvin-Benson cycle in rice leaf using the ILSA system (Fig. 4, Additional file 1: Fig. S2). These ^13^C abundance of each photosynthetic metabolites together with the net photosynthetic rate measured by gas exchange were used to derive a metabolic flux map using isotopically nonstationary metabolic flux analysis (INST-MFA) (Fig. 4A, Additional file 5: Data S4). Our metabolic model includes 40 reactions, five of which are independent efflux (Additional file 4: Data S3). The residual sum of squares (RSS) of fitting is 9.20, which were accepted based on χ^2^ tests with degrees of freedom equal to 30 for our model. The modeled carboxylation flux to oxygenation flux ratio (3.38:1) was near to the previous report in *Arabidopsis thaliana* (3.5:1). While the photorespiratory flux (the flux of glycine decarboxylation) is 5.7% of net CO_2_ assimilation compared with 16.7% in *Arabidopsis thaliana*. Interestingly, there are 61.4% glycine exported from model while only 0.26% reported in *Arabidopsis thaliana*, indicated a wider function of glycine in rice [4]. Furthermore, the modeled starch synthesis flux to sucrose synthesis flux ratio is about 14.7:1 compared with 1:1.9 reported in *Arabidopsis thaliana* [15] (Fig. 4, Additional file 6: Data S5). These new findings from INST-MFA need to be tested later with independent methods. It is worth emphasizing here that so far there are two earlier reports on the application of INST-MFA on land plants [3, 29]. This scarcity of study using INST-MF can be attributed to the technical complexity of INST-MFA, the difficult to gain an accurate metabolic network and the shortage of methods to validate the obtained fluxes from INST-MFA [30].

### Comparison with earlier leaf sampling method

Leaf sampling for metabolic profiling in most earlier studies has been mainly performed manually [2, 12, 13, 24, 31]. As a result of the high sensitivity of metabolite concentrations to environmental perturbation, the variations introduced by manual operation of the sampling apparatus will create variations in the measured metabolite concentrations, which make the interpretation of the difference in the metabolite concentrations challenging and hence made it challenge to use such data for metabolic flux analysis.

Some custom-built chambers have been built to perform ^13^CO_2_ labelling experiments to support metabolic flux analysis [3, 15, 16, 27]. But the time interval of sampling is usually longer than 10 s [15]. Furthermore, during the sampling process, the requirement for a strict control of the air flow rate, light level, liquid nitrogen and other labelling conditions makes the sampling process rather technically challenging. With ILSA, all environmental settings can be set up prior to sampling and the whole sampling procedure can be automated, which minimize the manual involvement to decrease the possibility of error introduction. This not only improves the sampling accuracy, but also makes possible to have high-throughput sampling.

In Table 2, we compared the features of ILSA and two other published sampling protocol/tools. These three methods can control the environments inside the leaf chamber and leaf samples can be frozen with liquid nitrogen. ILSA has a number of advanced features, i.e. it can provide dynamic light conditions during labelling and sampling; it has a higher portability and extensibility; and it takes the least time for sampling among three methods. Directly punching leaves into liquid nitrogen is not only much faster than manually pouring of liquid nitrogen onto leaves, but also more efficient and reliable than freezing clamping. The rapid sampling speed of ILSA ensures that the perturbation on the photosynthetic metabolic status and the gas exchange signal can be minimized.

**Table 2:** Summary of ILSA and other protocol/tools

	Manual protocol [27]	Freeze clamping [26]	ILSA
Sampling mode	Manual	Pneumatic	Automatic
Freezing mode	Freezing with LN_2_		
Time of sampling (${\tau }_{s}$)	~ 10 s	0.1 s	0.05 s
^13^C turnover time	~ 3 s	N.A.	~ 0.60 s
Air flow	5 L min^−1^	1 L min^−1^	1–6 L min^−1^
Leaf chamber (area/volume)	39.5 cm^2^/380 mL culture box	8 cm^2^/~ 16 mL chamber	16 cm^2^/~ 30 mL semi-open chamber
[CO_2_] control	√	√	50–10,000 ppm
Dynamic light	×	×	√
Light intensity	External	0–2000 μmol·m^−2^·s^−1^	0–1200 μmol m^−2^ s^−1^
Humidity control	×	×	√
Portability	Embedded in workbench	Desktop	Independent and movable
Extensibility	Unavailable	Single use	Can be connected in series or parallel

### Applications of the ILSA

So far, most studies on photosynthetic fluxes focus on the steady state metabolic flux [3, 15, 16]. Though the theory on none-steady-state metabolic flux have been developed [32–34], dynamic flux analysis for photosynthetic tissues is hampered by the lack of an effective and fast method to sample ^13^C labeled leaf sample under controlled conditions. Based on Nyquist-Shannon sampling theorem, only when the time interval of sampling is less than the characteristic time of a system, can the data collected be used to faithfully describe the dynamics of a system. By being able to sample ^13^C labeled leaves within one second, ILSA described in this report can be used to study many fundamental questions on leaf physiology. Here we list three examples to illustrate the potential applications:

1. Studying the metabolic flux changes under different light and CO_2_ levels. Though photosynthetic properties of leaves under different light and CO_2_ levels have been intensively measured and modeled [35]. However, the model is based on an assumed flux distribution in the photosynthesis, which need to be tested experimentally.
2. Recent evidence suggest that the glycine and serine might exit the photorespiratory pathway [8]. However, how large is the flux and how they vary under different plants or under different conditions is still unclear.
3. There are large variations of metabolomics in C_3_ leaves [2]. However, what is the underlying flux variations that produce such variations in the metabolomics are largely unknown. Understanding the metabolic structure and also the flux distribution will help understand the responses of plant primary metabolism to metabolic or genetic manipulation.

## Conclusions

Here we report the design and application of a new device, the ILSA. ILSA equipped a semi-open chamber can implement the isotopic labelling of leaf under a controlled environment. Using the ILSA, the leaf sample could be freeze in 0.05 s, which the metabolic homeostasis would not be inferred during the sampling. With the ILSA, we characterized the pool-size change of many primary metabolites under dark accumulation in rice leaf. We also demonstrated a variation of metabolic flux map in different C_3_ species. Further application of the ILSA will facilitate study on photosynthetic metabolic profiling and metabolic flux, especially for several crops such as rice, maize and wheat.

## Methods

### Measurement of dynamic leaf photosynthesis rate

Rice cultivar IR64 was used to measure the dynamic leaf photosynthesis under changing light. The dynamic leaf photosynthetic rates after changing light levels were measured with infrared gas analyzer system LI-6400XT (LI‐COR, Lincoln, Nebraska, USA). Flag leaves were used in the measurement. During the measurements, we set a reference CO_2_ concentration of 400 ppm and a photosynthetic photon flux density (PPFD) initially at 1000 μmol m^−2^ s^−1^ for 10 min followed by a PPFD of 500 μmol m^−2^ s^−1^ for 30 mins. The dynamic changes of leaf photosynthetic rates were recorded every 1 s during the measurement.

### Estimating the time of photosynthetic metabolite sampling

During metabolomic profiling or metabolomic flux, the time of sampling needs to be sufficiently short to avoid samples being significantly affected. We treat leaf photosynthetic metabolism as a black box and used the analytical methods of linear time-invariant systems (LTI systems) for the analysis [36]. This essentially treats photosynthetic metabolism as a LTI system during a short period of day. A LTI system has a characteristic time, which can be used to calculate the theoretical upper limit of the sampling time that can be allowed to ensure sufficiently high-quality data. First, according to the Nyquist-Shannon sampling theorem, the time interval of sampling (${{\varvec{\tau}}}_{{\varvec{i}}}$) should be at most 1/2 of the system characteristic time (${{\varvec{\tau}}}_{{\varvec{p}}}$) to obtain all the information for a LTI system. If so, the change of system can be approximated as a linear change without losing information of system:

5 $${{\varvec{\tau}}}_{{\varvec{i}}}\le \frac{{{\varvec{\tau}}}_{{\varvec{p}}}}{2}$$

Second, time interval of sampling (${{\varvec{\tau}}}_{{\varvec{i}}}$) includes two parts: sampling and waiting. Because leaf photosynthesis can be affected during the time of sampling, the percentage of sampling time in the time interval should be as small as possible. In this study, 5% is used as the maximal ratio between time of sampling (${{\varvec{\tau}}}_{{\varvec{s}}}$) and the time interval of sampling (${{\varvec{\tau}}}_{{\varvec{i}}}$).

6 $$\frac{{{\varvec{\tau}}}_{{\varvec{s}}}}{{{\varvec{\tau}}}_{{\varvec{i}}}}\le 5\%$$

### Design and implementation of ILSA

#### Framework of apparatus

The system integrates a gas mixer, semi-open leaf chamber and a freezing and sampling apparatus. The freezing and sampling apparatus includes three functional parts, which are linked together to a programmable logical controller (PLC). Gas mixer and PLC are assembled individually, while the semi-open leaf chamber and the freezing and sampling apparatus are assembled as one integrated unit. The number of the ILSA sampling units can be customized, and the maximum number of units depends on the performance of PLC in use. In our demo ILSA, 4 such units are integrated as one sampling array (Fig. 2A, E). The system uses 220 V DC power to drive the PLC, light source, mass flow controller (MFC) and other electronic components. An external high-pressure gas pump (> 0.8 MPa) is used to drive freezing and sampling mechanism. The controlling program with a graphical user interface (GUI) monitoring the sampling progress is used to connect to the ILSA by USB port. Because of the compact design, the turnover time of gas inside the chamber is only 0.63 s (Table 2), which is short compared to 3 s in previous report [27]. So, we did not reserve extra time to exchange chamber CO_2_. Semi-open chamber of ILSA can contain 2 or 3 rice leaves in parallel. Since in one single labelling experiment 4 chambers can be used, we can sample 4 time points after labelling and get 2–3 biological samples for each time point. Technically, ILSA can support 16 sampling units connected either in parallel or in series. All labelling and sampling operations are automated by the program. To minimize the interference of liquid nitrogen on the leaves, the liquid nitrogen in the sampling box needs to be manually added 10 s before sampling.

#### Gas mixer

Two mass flow controllers (MFC) are linked with external gas supply. One MFC with a range of 400 standard liter per minute (SLM) is linked with a mixing gas (79% N_2_, 21% O_2_). The other MFC with a range of 30 standard cubic centimeter per minute (SCCM) linked with a three-way solenoid valve is used to switch ^12^CO_2_ and ^13^CO_2_ gases. The flow-controlled mixing gas passes through a humidity adjustment pipe. The humidity-adjusted air converges with either ^12^CO_2_ or ^13^CO_2_ and is then transported into each semi-opened leaf chamber.

Because the CO_2_ concentration of the ambient air is around 400 ppm, the air flow of CO_2_ is extremely lower than N_2_ and O_2_. To reduce the time of replacing ^12^CO_2_ by ^13^CO_2_, the CO_2_ air path needs to be as short as possible. Furthermore, since 0.04% additional air flow will not have a significant impact on the humidity of the overall airflow. So, the humidity of air is adjusted before mixing with CO_2_ in ILSA.

#### Semi-opened leaf chamber

The leaf chamber is covered by two replaceable cellophanes both on the top and at the bottom (Fig. 2C). Air is pumped into the chamber from both the upper and lower sides simultaneously. Before sampling, leaf chamber is closed and isolated from the outside air. During sampling, hammer with sharp edges is automatically controlled to cut off cellophanes and the leaf inside a chamber rapidly and push them all into liquid nitrogen (Fig. 2B). The average sampling time (${{\varvec{\tau}}}_{{\varvec{s}}}$) is 52.64 ms (± 8.03 ms, S.D. N = 360). After sampling, the cellophane is cut so the leaf chamber is exposed to ambient air. Before the next sampling, a new cellophane is installed on the leaf chamber. The gas outlet of each unit is linked with a rotameter to measure the outward flux. Considering a standard air flow rate of around 2 L min^−1^ and a chamber’s volume of 28.8 cm^3^, a theoretical turnover time for ^13^CO_2_ to replace ^12^CO_2_ is about 0.6 s for ^13^CO_2_ labelling [27].

#### Light source

Two light sources are placed above the chamber (Fig. 2B). The illumination angle is adjusted to fully cover the whole cellophane area of the chamber. Each light source consists of a semiconductor lamp bead and equips with two cooling fans. PAR external chamber sensor of LI-COR6400XT is used to calibrate the light level on the chamber. Linear range of light is 50 to 1200 μmol m^−2^ s^−1^.

#### Freezing and sampling mechanism

To terminate metabolic reactions of a leaf as fast as possible, a custom-built high-speed cylinder is used to pull the freezing hammer down to a semi-open chamber (Fig. 2B). The cylinder stroke is 100 mm long, and the speed is up to 2 m s^−1^ which means that the freezing hammer can be pull down by 100 mm in 0.05 s. The freezing hammer is positioned directly above the leaf chamber and driven by a cylinder at the bottom. The freezing hammer is hollow inside, which can be filled with dry ice for pre-cooling before sampling.

### High performance liquid chromatography—triple quadrupole MS

In the 2nd top fully expanded leaf of each plant, the segment 1/3 from the leaf tip was sampled for metabolic profiling. All leaf samples were transferred rapidly into a pre-frozen 2 mL EP tube after ILSA sampling and stored in liquid nitrogen for metabolite extraction. After grinding, each sample was dissolved with 800 μL extraction buffer (methanol: chloroform = 7:3 (v/v), − 20 ℃ pre-cooling) and incubated under – 20 ℃ for 3 h. Then 560 μL distilled water (ddH_2_O) was added and mixed with each sample, 800μL supernatant was extracted after centrifugation (×2200*g*, 10 min, 4 ℃). After that, 800 μL buffer (methanol: ddH2O = 1:1(v/v), 4 ℃ pre-cooling) was mixed with sample for another extraction. For each sample, 1.6 mL supernatant was filtered with 0.2 μm nylon filter. Among them, 1 mL was used for high performance liquid chromatography—triple quadrupole MS analysis (HPLC–MS/MS) following [2, 15]. 20 μL was used for quality control (QC) sample. All extraction procedures were performed on ice.

Luna NH_2_ column (3 μm, 100 mm × 2 mm, Phenomenex co. Ltd, USA) was used for liquid chromatography (LC). Eluent A: 10 mM Ammonium acetate and 5%(v/v) acetonitrile solution, adjusted to pH 9.5 with ammonia water. Eluent B: acetonitrile. The following gradient was used for elution: 0–1 min, 15% A; 1–8 min, 15–70% A; 8–20 min, 70–95% A; 20–22 min, 95% A; 22–25 min, 15% A. During the mass spectrometry analysis, QTRAP 6500 + (AB Sciex, co. Ltd, USA) was used in MRM model, all parameters used followed [2, 15] with optimization (Additional file 2: Data S1). Concentration of all metabolites in samples were calculated based on the “concentration-peak area” curve of standard samples and converted to nmol g^−1^ FW with specific leaf weight which measured previously.

### Metabolic flux analysis

We used ILSA to take leaf samples at 0, 50, 100, 150, 200, 250, 300, 350, 450 and 500 s after ^13^CO_2_ (> 99%, Cambridge Isotope Laboratories, USA) is used. CO_2_ concentration in ILSA semi-open chamber was set to 500 ± 50 ppm, photosynthetic photon flux density (PPFD) used was 1200 μmol m^−2^ s^−1^. Before labelling, all leaves have been pre-illuminated at the same condition for 30 min. Metabolite isotopmers were extracted and measured with HPLC–MS/MS as described in the above section. The metabolic pathway model of [15] (Additional file 4: Data S3) was used. The model was fitted with the mean ^13^C abundance of three biological individuals with Open-Mebius [37].

## Supplementary Information

**Additional file 1: Figure S1.** Comparison of metabolite concentrations with previous report (Arrivault, et al., 2019). N = 6 for ILSA, N = 8 for reference (Arrivault, et al., 2019). Error bar indicated the S.E.M. **Figure S2**. Dynamic isotope labeling trajectories of measured metabolites. Data point with error bar (S.D. N = 3) represent the measured value, line represent the INST-MFA fitted result. M0, M1, M2, … means the mass isotopomer of each metabolites with certain number of 13C. [M6] + isotopomer of ADPG and UDPG are not be included because of the lack of labeling.

**Additional file 2: Data S1.** HPLC–MS/MS parameters used for metabolic profiling and ^13^C abundance of metabolites.

**Additional file 3: Data S2.** Metabolic profiling of IR64.

**Additional file 4: Data S3.** Metabolic network used for metabolic flux analysis.

**Additional file 5: Data S4.** 13C labelling data of IR64.

**Additional file 6: Data S5.** Calculated photosynthetic flux of IR64.

## Data Availability

All data generated or analyzed during this study are included in this published article and its additional files.

## Abbreviations

2PG: 2-Phosphoglycolate
ADPG: Adenosine diphosphate glucose
DPGA: Bisphosphate-glycerate
E4P: Erythrose-4-phosphate
F6P: Fructose-6-phosphate
FBP: Fructose-1,6-bisphosphatase
GLX: Glyoxylate
Gly: Glycine
PenP: Pentose phosphate
PEP: Phospho*enol-*pyruvate
PGA: Phosphoglycerate
RuBP: Ribulose 1,5-bisphosphate
S7P: Sedoeheptulose-7-phosphate
Ser: Serine
T3P: Triose 3-phosphate
UDPG: Uridine diphosphate glucose
