# Supplementary figures and images for: An integrated isotopic labeling and freeze sampling apparatus (ILSA) to support sampling leaf metabolomics at a centi-second scale
Source: Plant Methods. 2022 Jul 30;18:97. doi: 10.1186/s13007-022-00926-7 (PMC9338585; doi:10.1186/s13007-022-00926-7)

Photosynthetic metabolites in rice(IR64)

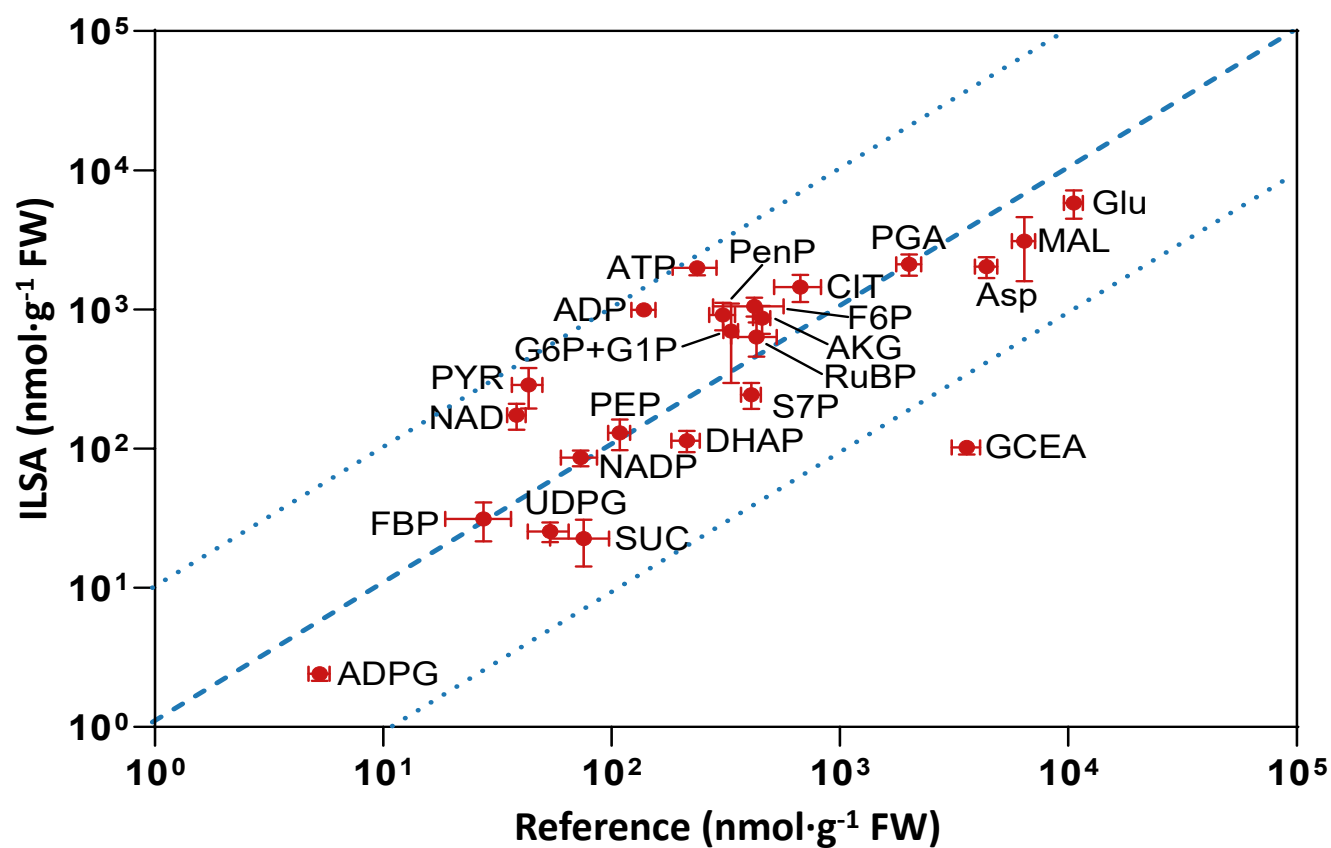

Figure S1.

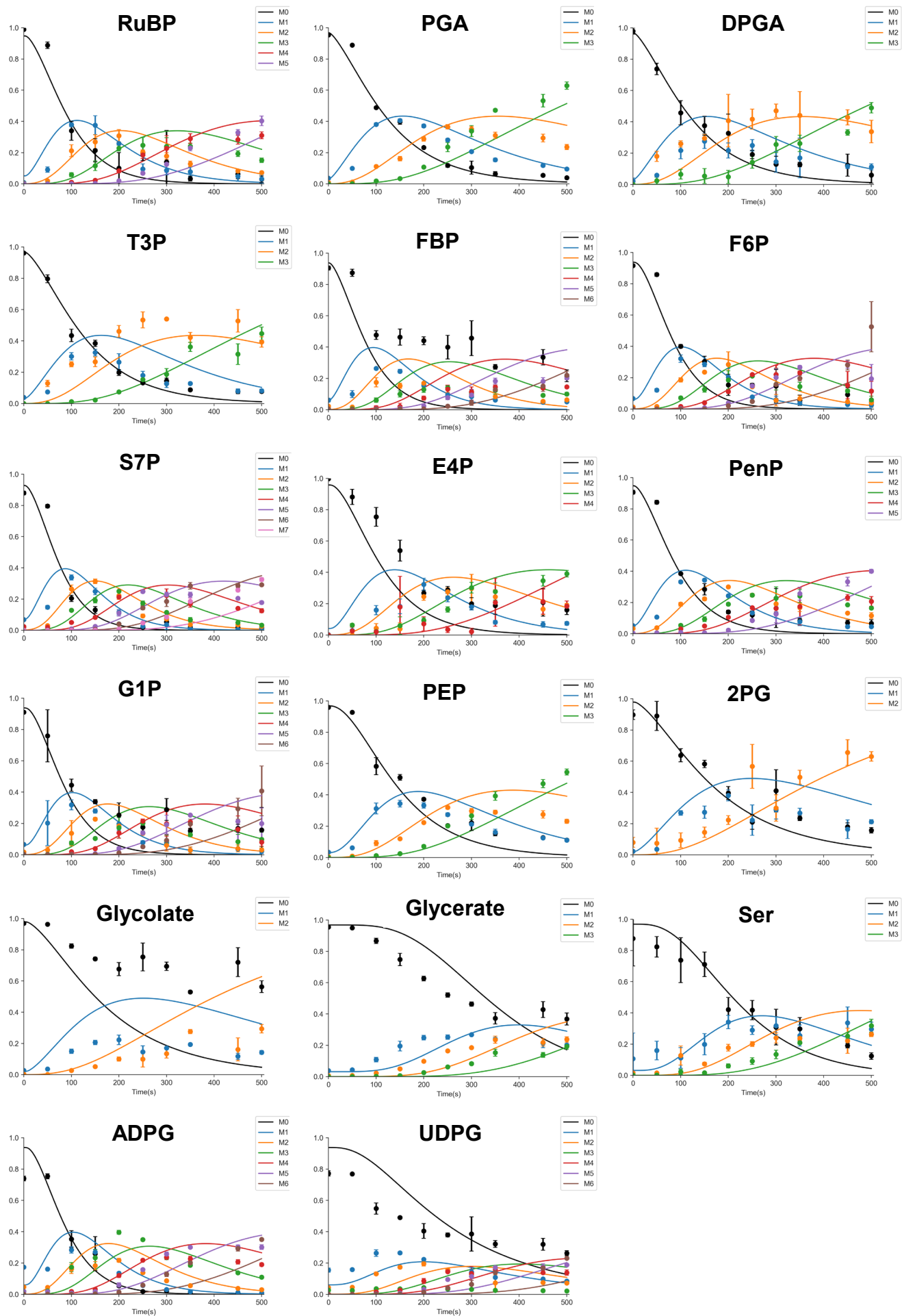

Figure S2.

Supplement: Supplementary file 1 — Additional file 1: Figure S1. Comparison of metabolite concentrations with previous report (Arrivault, et al., 2019). N = 6 for ILSA, N = 8 for reference (Arrivault, et al., 2019). Error bar indicated the S.E.M. Figure S2. Dynamic isotope labeling trajectories of measured metabolites. Data point with error bar (S.D. N = 3) represent the measured value, line represent the INST-MFA fitted result. M0, M1, M2, … means the mass isotopomer of each metabolites with certain number of 13C. [M6] + isotopomer of ADPG and UDPG are not be included because of the lack of labeling. [file 13007_2022_926_MOESM1_ESM.pdf]
